# Self-Standing Carbon Fiber Electrodes Doped with Pd Nanoparticles as Electrocatalysts in Zinc–Air Batteries

**DOI:** 10.3390/molecules30122487

**Published:** 2025-06-06

**Authors:** Cristian Daniel Jaimes-Paez, Miguel García-Rollán, Francisco José García-Mateos, Ramiro Ruiz-Rosas, Juana M. Rosas, José Rodríguez-Mirasol, Tomás Cordero, Emilia Morallón, Diego Cazorla-Amorós

**Affiliations:** 1 Departamento de Química Física, Instituto Universitario de Materiales de Alicante (IUMA), University of Alicante, Ap. 99, 03080 Alicante, Spain; cristian.jaimes11@ua.es; 2 Departamento de Ingeniería Química, Andalucía Tech, University of Malaga, Campus de Teatinos s/n, 29010 Malaga, Spain; mgarciarollan@uma.es (M.G.-R.); garciamateos@uma.es (F.J.G.-M.); jmrosas@uma.es (J.M.R.); mirasol@uma.es (J.R.-M.); cordero@uma.es (T.C.); 3 Departamento de Química Inorgánica, Instituto Universitario de Materiales de Alicante (IUMA), University of Alicante, Ap. 99, 03080 Alicante, Spain; cazorla@ua.es

**Keywords:** carbon fibers, lignin, electrospinning, electrocatalyst, ORR, Zn-air battery

## Abstract

In this work, the effect of the palladium precursor on the Oxygen Reduction Reaction (ORR) performance of lignin-based electrospun carbon fibers was studied. The fibers were spun from a lignin-ethanol solution free of any binder, where different Pd salts were added at two concentration levels. The system implemented to perform the spinning was a coaxial setup in which the internal flow contains the precursor dispersion with the metallic precursor, and ethanol was used as external flow to help fiber formation and prevent drying before generating the Taylor cone. The obtained cloths were thermostabilized in air at 200 °C and carbonized in nitrogen at 900 °C. The resulting carbon fibers were characterized by physicochemical and electrochemical techniques. The palladium precursor significantly affects nanoparticle distribution and size, fiber diameter, pore distribution, surface area and electrochemical behavior. The fibers prepared with palladium acetylacetonate at high Pd loading and carbonized at 900 °C under a CO_2_ atmosphere showed high mechanical stability and the best ORR activity, showing near total selectivity towards the 4-electron path. These features are comparable to those of the commercial Pt/C catalyst but much lower metal loading (10.6 wt.% vs. 20 wt.%). The most promising fibers have been evaluated as cathodes in a zinc–air battery, delivering astonishing stability results that surpassed the performance of commercial Pt/C materials in both charging and discharging processes.

## 1. Introduction

Carbon materials, with their dimensional diversity (ranging from 0D to 3D) [1,2], their versatility in terms of structure, low cost, accessibility and abundance in the earth crust, have been highlighted as suitable materials for a wide range of applications, including catalysis, electronics and energy storage and generation [3,4,5,6]. Specifically, their use as electrodes has gained attention due to their chemical stability, their ability to be used at high temperatures and the ease of modifying their surface chemistry [2].

Carbon fibers are characterized by their exceptional mechanical strength, electrical and thermal conductivity, as well as their high specific surface area [7]. These properties have led to an extensive study of carbon fibers due to their numerous potential applications. Their manufacture can be achieved through various techniques [8,9,10,11].

In this context, lignin, a natural biopolymer present in plants, has emerged as an abundant and renewable material, proposed as a precursor to produce carbon fibers. Its richness in aromatic and phenolic groups makes it especially suitable as a precursor material for the synthesis of carbon materials [12]. Lignin is also a well-known precursor for the preparation of carbon submicron fibers and nanofibers using the electrospinning technique [13,14,15,16,17,18].

Electrospinning, a technique that uses an electric field to draw liquids into fine fibers, has proven to be a versatile and economical option for preparing carbon fibers with a wide range of diameters and morphological properties [19,20,21]. Furthermore, this technique allows obtaining a uniform dispersion of metals in the fibers, which, after thermal stabilization and carbonization, can result into electrocatalysts based on carbon fibers with an excellent dispersion of metal nanoparticles [11,22,23,24]. Another interesting advantage arises from the non-woven mat structure of electrospun carbon fibers, resembling that of gas diffusion layers, allowing to cast them into electrochemical devices as self-standing electrodes [25,26,27].

Metallic nanoparticles, particularly noble metal nanoparticle-modified electrodes, have demonstrated high electrocatalytic activity, especially in energy conversion reactions such as oxygen reduction reaction (ORR). This reaction is essential in promising technologies such as zinc–air batteries and fuel cells [28,29,30,31,32]. Zinc–air batteries are particularly attractive due to their high theoretical energy density (1086 Wh kg^−1^) and established manufacturing maturity [33,34,35]. In this context, palladium has emerged as a valuable dopant in electrocatalyst materials. Its unique electronic properties enhance the efficiency and stability of these batteries, offering a promising alternative to traditional platinum-based catalysts. Moreover, Pd nanoparticles used to dope materials such as carbon fibers have shown effectiveness as electrocatalysts in applications related to energy storage and conversion [36,37,38,39,40,41].

Previous studies, including our own, have highlighted the potential of Pd-doped carbon fibers as ORR electrocatalysts [27,42]. Nevertheless, despite their promising potential as ORR electrodes, several critical challenges remain: the influence of the Pd precursor on the structure, distribution and oxidation state of nanoparticles loaded on lignin-based electrospun carbon fibers is not yet fully understood. Moreover, the correlation between key synthesis parameters such as stabilization time and carbonization atmosphere remains to be explored. Also, achieving an optimal porous structure that maximizes the exposure of active sites while maintaining favorable conductivity continues to be a significant challenge.

To address these limitations, in this study, Pd nanoparticles are incorporated into lignin-derived carbon fibers via electrospinning without additional additives, aiming to combine the catalytic benefits of Pd with the structural advantages of carbon fibers. A key distinction in this study lies in the exploration of different Pd precursors and loading strategies to enhance catalytic performance. We present a detailed study of the physicochemical properties of the resulting electrocatalysts to identify the most suitable candidate for ORR. Finally, we evaluate the performance of the most promising Pd-containing carbon fiber in a zinc–air battery, employing a self-standing electrode design that avoids additional binders and substrates. This approach simplifies the assembly process and improves mechanical flexibility, allowing for efficient electron transfer and paving the way for future applications in flexible energy storage devices.

## 2. Experimental

### 2.1. Electrocatalyst Preparation

The electrocatalysts have been prepared following a similar methodology used in previous works [11,23,24,27], using lignin, ethanol and palladium precursor salts (palladium acetate (Ac), palladium acetylacetonate (AcAc) or palladium chloride (Cl) (Sigma Aldrich, Darmstadt, Germany). Lignin was obtained through the organosolv process (Alcell^®^) (Repap Technologies Inc., Vancouver, BC, Canada) and it was mixed with ethanol in a 1:1 mass ratio. The different Pd salts were added to the lignin/ethanol solution under Pd/lignin mass ratios of 0.0075 and 0.015 corresponding to a nominal Pd concentration in the carbon fiber electrocatalysts of ca. 2.5 wt% (L) and ca. 5 wt% (H).

Electrospinning was conducted using a coaxial setup under controlled voltage and flow conditions, optimized for each Pd precursor. The fibers underwent thermostabilization in air at 200 °C, with a heating rate of 5 °C h^−1^, using different holding times. The stabilized fibers were carbonized under nitrogen, reaching 900 °C. Additionally, the effect of air stabilization time and of using a CO_2_ atmosphere during thermal treatment was assessed (for the detailed electrocatalyst preparation see Supporting Information).

The nomenclature used in this work indicates the Pd precursor used (Ac, AcAc or Cl), followed by the Pd concentration (L or H) for carbon fibers stabilized for 48 h and carbonized in N_2_ at 900 °C. The samples prepared modifying the air stabilization time or the thermal treatment atmosphere are denoted by adding 12 h and CO_2_ at the end of their names, respectively.

### 2.2. Physicochemical Characterization

The surface morphology and structure of the metal-loaded carbon fibers were analyzed by scanning electron microscopy (SEM), using a JSM 6490LV (JEOL, Tokyo, Japan) microscope, working at 20 kV and transmission electron microscopy (TEM), with a Talos F200X (Thermo Fischer Scientific, Waltham, MA, USA) microscope at 200 kV, respectively. The average Pd particle size and particle size distribution were estimated using ImageJ software/V 1.53K) measuring the size of, at least 100 Pd particles of every carbon fiber.

In order to determine the porosity of the carbon fibers, nitrogen adsorption–desorption isotherms at −196 °C were performed in an ASAP2020 apparatus (Micromeritics, Norcross, GA, USA). The samples were degassed at 150 °C for 8 h under vacuum. The surface apparent area (A_BET_) was determined with the BET equation using the data obtained from the N_2_ adsorption–desorption isotherms. The alpha-s method was used to evaluate the micropore volume (V_micro_), being the mesopore volume (V_mes_) obtained by the subtraction of this micropore volume to the total pore volume at a relative pressure of 0.98. The micropore size distributions were calculated by using the SAIEUS program (V 3.0), 2D-NLDFT heterogeneous surface model was applied to the N_2_ adsorption isotherms [43].

X-ray photoelectron spectroscopy (XPS) on a PHI 5000 VersaProbe II equipment (Chigasaki, Japan), working with a monochromatic Al Kα radiation source (1486.6 eV), was used to study the surface chemistry of the metal-loaded carbon fibers. The peaks were corrected using the maximum of the C1s peak, which has been positioned to the XPS photoemission energy of the C-C and C=C bonds (284.5 eV).

The final Pd loading on the carbon fibers was analyzed by thermogravimetry analysis (STA200. Hitachi, Tokyo, Japan). Samples of 15 mg were calcined at 900 °C using a heating rate of 10 °C min^−1^. The metal loading was determined from the resulting ashes considering that palladium is in form of oxide. The experiments were replicated three times.

Finally, the Raman spectra data were acquired at 532 nm with a Jasco NRS-5100 spectrometer (Tokyo, Japan). X-ray diffraction (XRD) patterns of the electrocatalysts were recorded in a Bruker D8 Advance diffractometer (Billerica, MA, USA). The XRD patterns were measured using a Cu Kα radiation source and crystallographic structure assignments were made from the information presented in the Powder Diffraction File (PDF) database. Scherrer’s equation was used to determine the size of the crystallites.

### 2.3. Electrochemical Characterization

Electrocatalytic activity analysis was carried out using an Autolab PGSTAT302 potentiostat with SCAN25 module (Metrohm, Barendrecht, The Netherlands) with a current measurement accuracy of ±0.2%. The counter electrode was graphite, and the reference electrode was a Reversible Hydrogen Electrode (RHE), which was immersed in the working electrolyte through a Luggin capillary. The working electrode used was a rotating ring-disk electrode (RRDE) from Pine Research Instruments (Durham, NC, USA) with a glassy carbon disk electrode (diameter 5 mm) and an attached platinum ring.

For the electrochemical studies, the carbon fibers were ultrasonically dispersed to create a catalytic ink containing 1 mg of carbon fibers and 1 mL of a solution composed of 0.02% by volume of Nafion^®^ (Sigma-Aldrich, Saint Louis, MO, USA) and 20% by volume of isopropanol in water. Then, 120 µL of this dispersion was deposited on the glassy carbon disk. The materials were electrochemically characterized in 0.1 M KOH electrolyte under an inert nitrogen atmosphere by cyclic voltammetry (CV) over a potential range of 0 to 1.4 V (vs. RHE) at a sweep rate of 50 mV s^−1^. Several cycles were performed before the main measurement to activate and clean the catalytic sites.

Oxygen reduction reaction was measured by linear sweep voltammetry (LSV) tests at 5 mV s^−1^ over a potential range of 1.0 to 0 V (vs. RHE) in alkaline media, with oxygen saturation, at different rotational speeds ranging from 400 to 1600 rpm. During all tests, a constant potential of 1.5 V was maintained at the platinum ring electrode. The electron transfer number, denoted as “*n*”, was determined from the currents, in absolute value, generated at the disk (I_D_) and ring (I_R_) electrodes as follows [44]:

(1) $$n=\frac{4I_{D}}{I_{D}+I_{R}/0.25}$$

The collection efficiency of the RRDE, determined experimentally, was 0.25.

The Electrochemical Surface Area (ECSA) is estimated from the double-layer capacitance (*Cdl*). To calculate it, CV curves obtained in a potential range of 0.8–0.9 V vs. (RHE) were used for the Pd-doped carbon fibers at sweep rates of 10–100 mV s^−1^. The value of *Cdl* was determined at 0.85 V vs. RHE [27].

TOF was calculated using the current at 0.4 V in the LSV obtained in ORR, and the amount of metal was determined from XPS, assuming that this corresponds to the accessible metal. For the TOF calculations, the equations presented in a previous study have been used [27].

Impedance tests were carried out at a potential of 0.2 V vs. RHE using N_2_-saturated 0.1 M KOH. The frequencies ranged from 6000 to 0.1 Hz. The amplitude of the applied voltage (ΔE) during the EIS measurements was set to 0.01 (Vtop), and the RMS amplitude was not activated during the experiments.

A stability test was carried out by subjecting the samples to 500 cycles in a potential range between 0.5 and 1.0 V, at a sweep speed of 100 mV s^−1^, in an O_2_-saturated atmosphere.

### 2.4. Zinc–Air Battery

**Figure 1:**
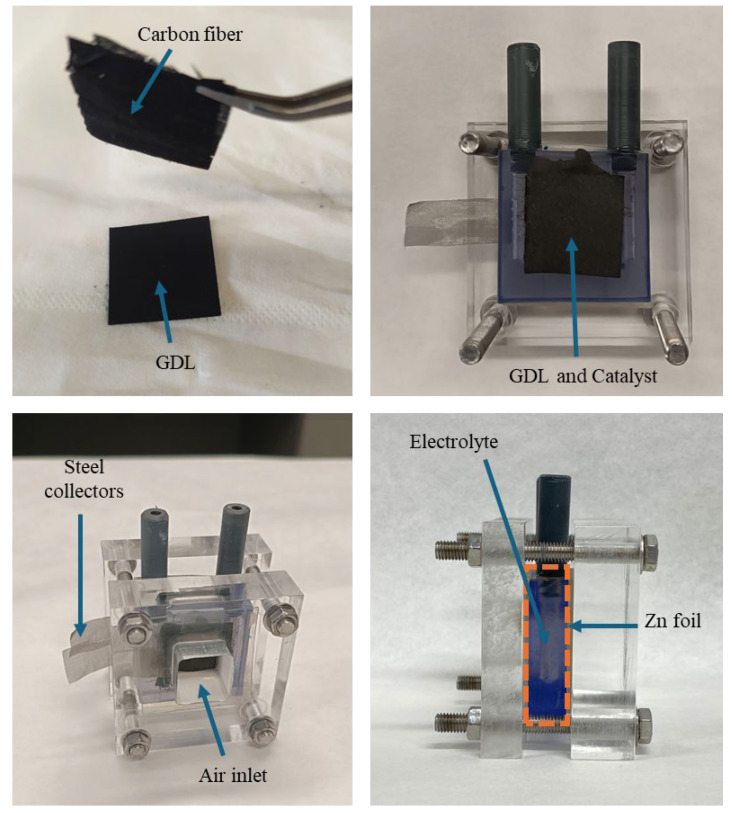
Zinc–air battery components.

The zinc–air battery used in this study was configured as shown in Figure 1. As the air (positive) electrode, the carbon fiber of the AcAcH-CO_2_ electrocatalyst fiber was used, which was placed directly on the gas diffusion layer (GDL) and was assembled into the system. For comparison purposes, the commercial Pt/C catalyst ink was painted with a brush (with 20 wt% Pt and 5% Nafion^®^ in isopropanol) on the gas diffusion layer (carbon paper, QUINTECH, Freudenberg H23C6, Indiana, PA, USA) with a surface loading of 1.3 mg cm^−2^. The negative electrode consisted of a polished zinc foil, and the electrolyte was prepared using an aqueous solution of 6 M KOH and 0.2 M Zn(O_2_CCH_3_)_2_. The geometric surface area of the electrodes was 2.8 cm^2^. Battery tests were carried out using an ARBIN SCTS multi-channel battery test potentiostat (College Station, TX, USA), with an error in the recorded currents of ±0.02 %. Polarization curves were generated at a rate of 1 mA s^−1^. In each 10 min cycle, galvanostatic charge and discharge cycles were performed at a constant current density of 1 mA cm^−2^. In addition, tests were carried out to evaluate the capacity of the battery and the cyclability was studied by subjecting it to different current densities.

## 3. Results and Discussion

### 3.1. Carbon Fibers Preparation and Characterization

The electrospinning of Alcell lignin solutions produces flexible non-woven mats that can be easily handled for the required application. Owing to the low glass transition temperature of technical lignins, electrospun lignin fibers require a thermostabilization stage, prior to the carbonization process, to increase the glass transition temperature through the generation of oxygen-containing species on the surface of the mats, thus avoiding its melting in the carbonization step [15].

The preparation yields are shown in Table 1. The thermostabilization step produced a noticeable decrease in the sample weight, associated with the evaporation of the ethanol and the condensation and dehydration reactions associated with the crosslinking of polymeric chains of lignin, which increases the glass transition temperature [11]. The addition of Pd into the lignin solution increases the loss of weight during this step due to the catalytic activity of Pd for oxidation reactions [23]. Among the explored Pd-precursors, palladium acetyl-acetonate delivers the lowest stabilization yield for both low and high metallic loadings in the solution. This decrease in the stabilization yields can be related to the decomposition of palladium acetyl acetonate, which occurs at 185 °C, close to the final stabilization temperature (200 °C) [45]. The thermostabilization was also carried out for a decreased time of 12 h in the AcAcH sample. As expected, a shorter holding time at 200 °C noticeably decreases the loss of weight during air stabilization (61% vs. 37%).

Table 1 also includes the carbonization yields of carbon fibers, which ranged from 30 to 50%, depending on the palladium content of the sample and the metal precursor used. The carbonization of the stabilized fibers leads to the release of volatile matter and the thermal decomposition of the functional groups of lignin, including those formed during the thermal stabilization process [46]. Another factor contributing to the weight loss in this stage is the decomposition of the chlorides, acetates and remaining acetylacetonate anions found in the Pd precursors. Note that the increase in Pd content produces a modification on the carbonization yield that depends on the nature of the precursor. Unlike the Cl and Ac systems (higher Pd content reduces carbonization yield), the AcAc system shows an increase when moving from low (AcAcL) to high (AcAcH) Pd content. This anomalous trend in the AcAc system can be attributed to the early decomposition of palladium acetylacetonate near the stabilization temperature, promoting enhanced crosslinking, as well as to the improved dispersion of smaller Pd nanoparticles in the AcAcH sample, which may favor carbon retention during pyrolysis. The carbonization of the AcAcH sample stabilized with a holding time of only 12 h rendered a lower yield (48% vs. 53%). However, the overall preparation yield was much higher (29% vs. 20%). If the carbonization atmosphere is replaced by CO_2_, the yield decreases down to 36%, achieving the lowest overall preparation value (13%), due to the gasification of the carbon matrix during the process.

The overall preparation yields (obtained by combining both the air stabilization and carbonization ones), show values similar to those previously reported for lignin-based electrospun carbon fibers loaded with 2 wt% of Pt or 10 wt% of Co using platinum acetylacetonate and cobalt nitrate, respectively, as metal precursors [23,24].

**Figure 2:**
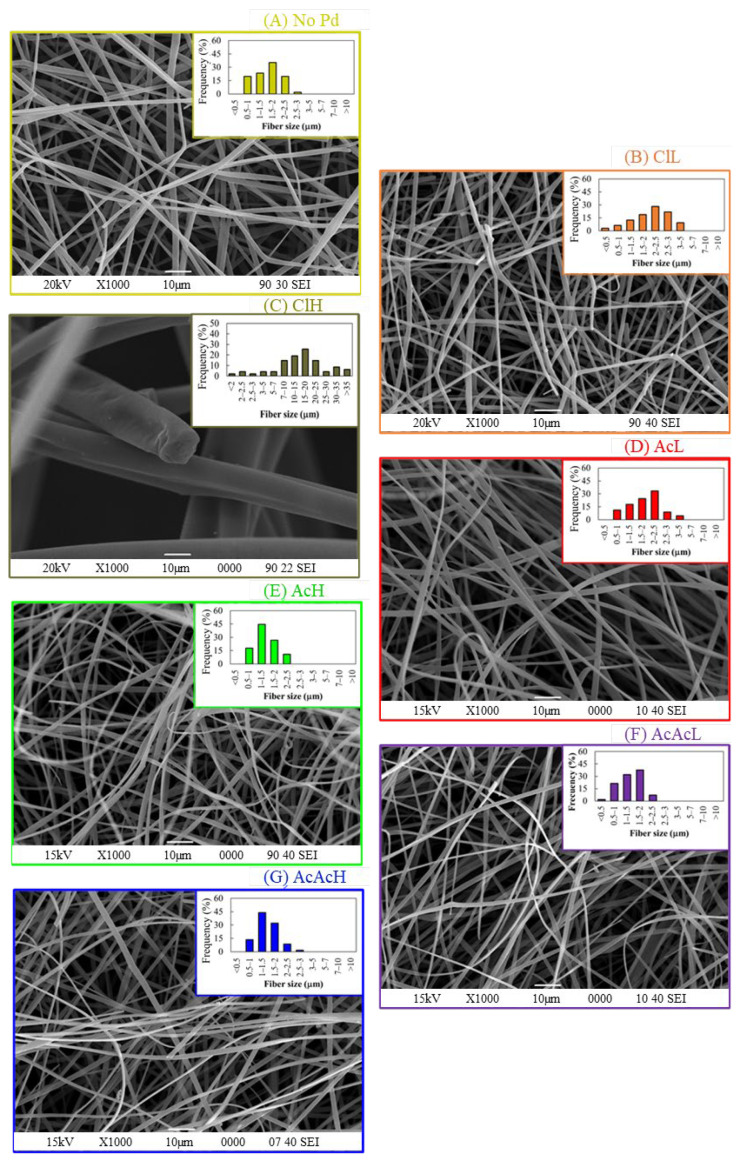
SEM images and fiber size distribution of the carbon fibers: (**A**) No-Pd, (**B**) ClL, (**C**) ClH, (**D**) AcL, (**E**) AcH, (**F**) AcAcL and (**G**) AcAcH.

Figure 2 shows the SEM images of the carbon fibers electrocatalysts. The Pd-loaded carbon fibers maintained their loose and fibrillar morphology. A slight modification of the average diameter of the carbon fibers is observed when metallic precursor is included in the lignin solution (Table 1). The increase in the electrical conductivity of the polymeric solution seems to allow the production of carbon fibers with lower average diameters. Specifically, the average size decreases from 1.6 µm for carbon fibers without palladium to 1.4 µm for ClL, AcAcL and AcAcH (Figure 2B, 2F and 2G, respectively). Conversely, the SEM image of the ClH sample (Figure 2C) shows much larger fiber diameter and broken fibers. The introduction of high concentrations of palladium chloride in the polymeric solution makes necessary the use of a high lignin solution flow rate, which prevents the formation of continuous lignin fibers, resulting in very short fibers and thicker carbon fibers that are hardly suitable for both catalytic and electrochemical applications.

**Figure 3:**
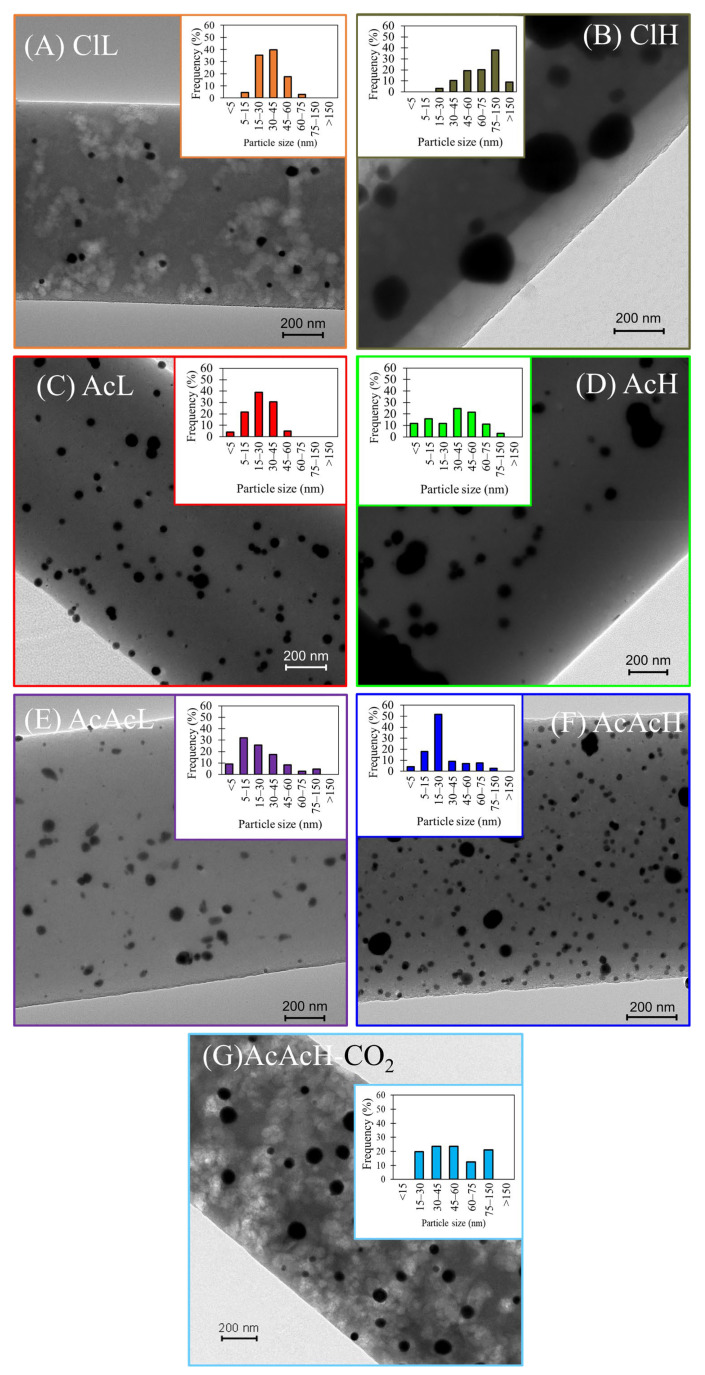
TEM images and Pd particle size distribution of the carbon fibers: (**A**) ClL, (**B**) ClH, (**C**) AcL, (**D**) AcH, (**E**) AcAcL, (**F**) AcAcH and (**G**) AcAcH-CO_2_.

Figure 3 shows TEM images of Pd-loaded carbon fibers. Carbon fibers with smooth and defect-free surfaces and well dispersed Pd nanoparticles are observed. The particle size distributions are noticeably modified by the selected metal precursor. Thus, with a low concentration of palladium chloride, a homogeneous distribution of the metal is observed in Figure 3A, with Pd particle sizes ranging from 5 to 75 nm, being the average particle size of about 35 nm (Table 1). The use of a higher Pd concentration delivers a widening of the Pd particle size distribution (PSD) up to 150 nm (Figure 3B), obtaining a mean particle size of 85 nm. The use of palladium acetate (Ac sample) favors the mobility of the metal particles, leading to the formation of large clusters. PSDs ranging from 5 to 60 nm and from 5 to 130 nm were obtained for AcL (Figure 3C) and AcH (Figure 3D), respectively. Accordingly, a higher concentration of the Pd salt increases the Pd average size from 38 to 60 nm. In contrast, the use of palladium acetylacetonate (Figure 3E,F) promotes a uniform distribution of metal particles mainly at high concentrations, resulting in average particle sizes below 24–27 nm (Table 1). For the AcAcH-CO_2_ sample (Figure 3G), the formation of mesopores by carbon etching is confirmed, and a notable widening of the Pd particle size distribution is observed, showing some signs of metal sintering.

To further validate the structural properties and distribution of Pd nanoparticles in the carbon fiber electrocatalysts, HRTEM and TEM mapping analyses were conducted. Figure S1 in the Supplementary Material shows HRTEM images of the samples ClH (A), AcH (B), and AcAcH (C), alongside a TEM mapping of the AcAcH-CO_2_ sample (D). The HRTEM images reveal well-defined lattice fringes with interplanar spacings characteristic of Pd nanoparticles, confirming their successful incorporation into the carbon matrix. In the TEM mapping of the AcAcH-CO_2_ electrocatalyst, the elemental distribution of Pd and C demonstrates a homogeneous dispersion along the carbon fiber structure.

**Figure 4:**
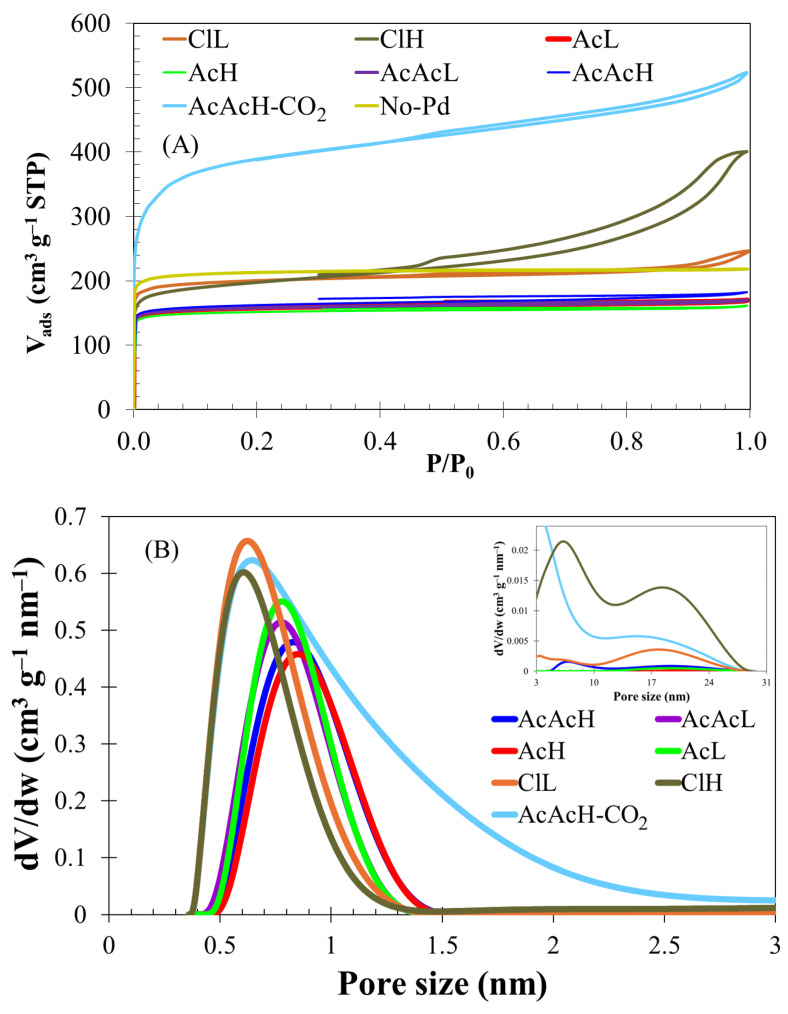
N_2_ adsorption–desorption isotherms at 196 °C (**A**) and Pore Size Distributions calculated from N_2_ adsorption desorption isotherm (**B**) of the carbon fibers.

Figure 4 plots the nitrogen adsorption–desorption isotherms at −196 °C and the pore size distribution of the different carbon fibers. The Pd-free carbon fibers exhibit a Type I isotherm according to the IUPAC, which is characteristic of a microporous material [47]. The use of different Pd precursors and the variation in the final Pd content have a strong influence on porosity. Specifically, Pd-containing carbon fibers prepared from palladium acetate (AcL and AcH) and palladium acetylacetonate (AcAcL and AcAcH) present a similar shape of the N_2_ adsorption isotherm. However, the incorporation of Pd produces a slight decrease in the porosity development, decreasing the micropore volume and the specific surface area values from 865 (in the absence of Pd) down to 615–650 m^2^ g^−1^. In contrast, the use of palladium chloride as a metal precursor enables much higher porosity development compared to its counterparts, especially in the case of ClH, where significant N_2_ adsorption at higher relative pressures and a type IV hysteresis loop between the adsorption and desorption branches is observed, Figure 4A. Mesopore volumes of 0.06 and 0.38 cm^3^ g^−1^ are obtained for the lower and higher Pd loadings in these samples, in clear contrast with the 0.01–0.03 cm^3^ g^−1^ observed for the rest of samples (Table 2). The high mesopore volume has been elsewhere related to the promoted generation of surface oxygen groups during the stabilization step. These functional groups are decomposed during the carbonization step, producing a selective gasification of the carbon fibers [23,24], as evidenced by the higher weight loss detected during the carbonization process when using PdCl_2_ as precursor, Table 1. The analysis revealed that the sample stabilized in only 12 h, presented a lower specific surface area (A_BET_ = 595 m^2^ g^−1^). This result underlines the relevance of the stabilization time in the development of porosity. As intended, the use of a CO_2_ atmosphere during carbonization at 900 °C for 2 h increased the porosity development, as the isotherm shows (sample AcAcH-CO_2_), reaching surface area values of 1460 m^2^ g^−1^, and doubling the micropore volume value, Table 2.

Figure 4B shows the pore size distribution of Pd carbon fibers prepared with different metal precursors. A bimodal pore size distribution is observed, centered in the micropore (0.4–1.4 nm) and mesopore (2–30 nm) ranges, for all the samples. For the micropore range, the increase in the metal content produces minor impacts, i.e., a slight decrease in the micropore volume, and widening in the pore size, while the use of palladium chloride delivers a lower pore size and a narrower PSD. Furthermore, in the case of AcAcH-CO_2_ the PSD broadens. The Pd precursor plays a more relevant role in the mesoporosity development; in addition, the CO_2_ treatment provides a greater range in the size of the porosity developed. While the development of mesoporosity is negligible when using acetate or acetylacetonate, the decomposition of palladium chloride allows the development of mesopores with sizes ranging from 5 to 27 nm, which is likely connected to the higher Pd particle size observed for this sample in TEM analyses.

Table 2 also presents the surface and bulk Pd content measured by XPS and thermogravimetric analysis, respectively. The oxidation state of palladium in carbon fibers seems not to be influenced by the metallic precursor (Figure S2), where a predominance of Pd^0^ at 335.2 eV was observed, in contrast to a very low presence of Pd^+2^ at 336.9 eV [48]. In contrast, the surface concentration is highly reliant on the nature of the precursor (Table 2). In the case of the carbon fibers prepared from palladium chloride (ClL and ClH) and palladium acetate (AcL and AcH), a notable disparity between surface and bulk Pd concentration values is observed, suggesting an uneven distribution of palladium, with Pd present mainly on the carbon fiber surface (Pd_XPS_ > Pd_TG_). In contrast, for the carbon fibers prepared from palladium acetylacetonate as metal precursor, a homogeneous Pd distribution is seen (similar surface and bulk Pd values, Table 2), in agreement with the results shown in the study of the TEM images of Figure 3. However, the Pd accessibility was negatively impacted by the use of a shorter air stabilization time, with sample AcAcH-12 h showing a surface Pd content of only 0.9 wt%. Similarly, prepared carbon fibers with such a low surface Pd amount showed decreased activity on methanol oxidation, making additional activation treatments necessary to increase the accessibility of the electrolyte to the active phase [23]. Consequently, the air stabilization time to favor the migration of Pd nanoparticles to the external surface of the fibers, enhancing the accessibility of O_2_ to the active sites, was set to 48 h. As a consequence of the CO_2_ treatment that produces the partial gasification of the carbon matrix the Pd bulk and surface contents increased by up to 10%.

**Figure 5:**
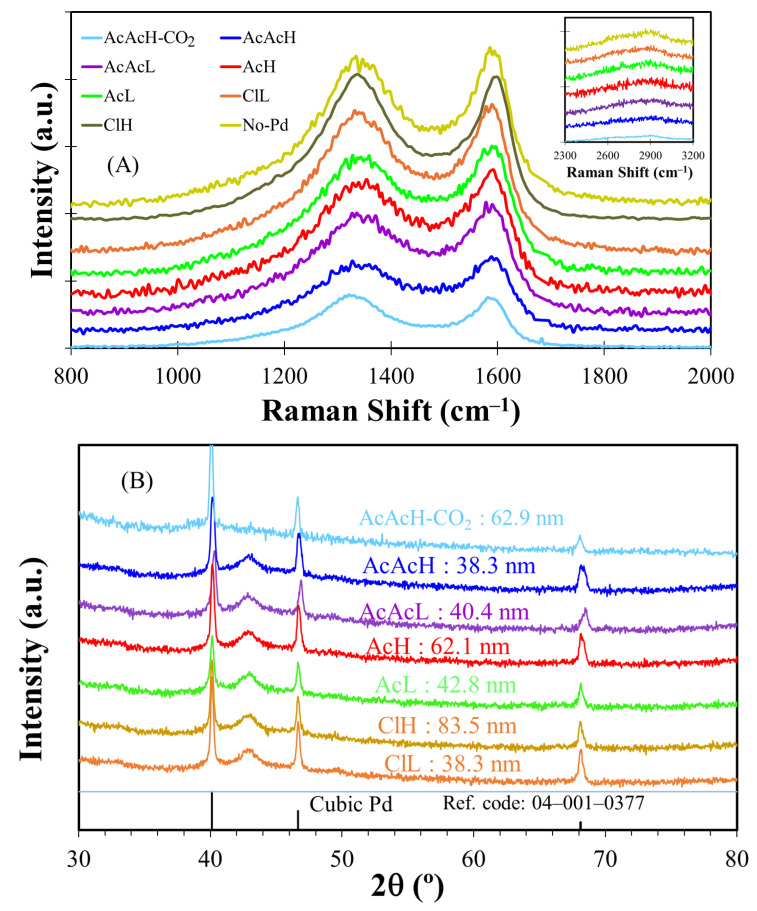
(**A**) first-order region of Raman spectra, second-order region inset and (**B**) XRD patterns of the electrocatalysts.

Structural information of the electrocatalysts has been obtained by Raman and XRD. The Raman spectra of the samples prepared with different precursors and metallic loadings are plotted in Figure 5A. The spectra show two broad and overlapping bands at around 1350 and 1590 cm^−1^, commonly described as the D and G bands, related to the presence of defects and ordered graphitic network, respectively [49]. A detailed deconvolution reveals the presence of two additional bands at ca. 1510 cm^−1^ and 1170 cm^−1^, the latter being related to surface oxygen groups and heteroatoms, such as palladium itself [50]. The detailed study of the area ratios, position, and width of G- and D-bands, Table S1, does not reveal relevant changes in structural order (i.e., crystalline sizes or stacking) for the analyzed samples, achieving values of the involved parameters in line with those reported in the absence of Pd [18] allowing to conclude that Pd does not affect the structural order of the carbonized fibers.

The XRD results of the Pd carbon fibers are shown in Figure 5B. Three peaks are observed at 40.11°, 46.66° and 68.12° for all the electrocatalysts, associated with the presence of the face-centered cubic phase of palladium (JCPDS card no. 05-0681) [51]. Figure 5B also includes the values of the palladium crystal sizes of each sample, calculated using the Scherrer equation. In general, Pd crystal size determined by XRD revealed an increase in Pd crystalline size at higher Pd concentrations, except for AcAcH. The use of the latter precursor also renders lower crystalline sizes than chlorides or acetates. Additionally, the presence of the characteristic carbon peak associated with the (100) plane at ~43° is observed in all the samples. However, in the CO_2_-activated sample, this peak appears significantly less pronounced. The decrease in the intensity of the (100) peak in the CO_2_-activated sample is likely related to a reduction in the average lateral size of the crystallites. This can be attributed to the preferential removal of carbon atoms at the edge positions of the graphene layers during the gasification process with CO_2_.

### 3.2. Electrochemical Characterization

**Figure 6:**
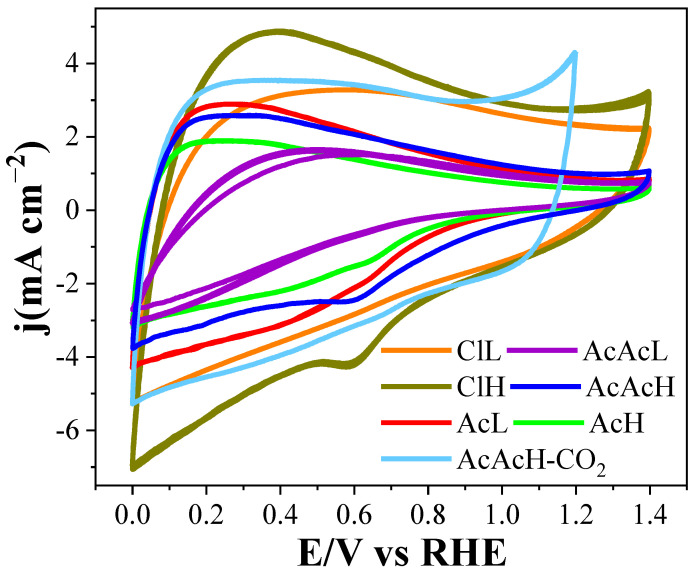
Cyclic voltammograms for the carbon fibers of different Pd precursors, scan rate of 50 mV s^−1^, N_2_ saturated, in 0.1 M KOH.

Figure 6 illustrates the cyclic voltammograms of the electrocatalysts studied in an alkaline medium at 50 mV s^−1^. To ensure stable material responses, each sample underwent 10 cycles within the 0 to 1.4 V. In the analyzed samples, capacitive behavior characteristic of carbon materials is observed. This behavior correlates with the area contained within the curves, and these areas seem to be aligned with the surface area developed by these materials (Table 2). In the case of carbon fibers derived from palladium chloride, it has been observed that they have a significantly enlarged BET surface area, which translates into an increase in the current observed in the voltammograms. This characteristic implies a greater capacitance that may facilitate electrochemical reactions, evidencing the direct influence of the active surface on the efficiency of the process. However, it is crucial to note that most of these voltammograms exhibit a pronounced tilt, implying some resistivity.

The pronounced capacitive response characteristic of porous carbon materials stands out, minimizing the observation of faradic processes linked to the presence of electroactive metals, such as palladium in this context. In some samples, a cathodic current peak at a potential of approximately 0.6 V during the negative sweep stands out, which is attributed to the reduction in the palladium oxide produced during the positive sweep at 1.4 V. Such electroactivity of the metals, located on the surface of the analyzed electrocatalysts, is overlapped with the double layer charge observed in the porous carbon fibers.

**Figure 7:**
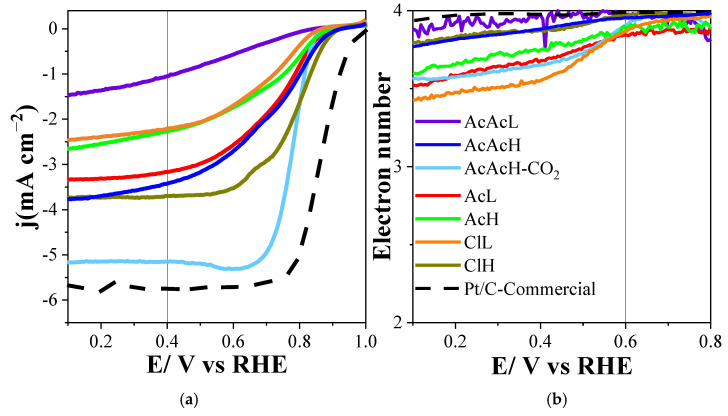
ORR performance in 0.1 M KOH, scan rate of 5 mV s^−1^, O_2_ saturated. (**a**) Linear sweep voltammograms; (**b**) Electron transfer number.

In the polarization curves used to evaluate the performance against ORR (Figure 7 and Table 3), it is observed that the electrocatalysts obtained from various palladium precursors consistently follow a 4-electron reaction path, with values always greater than 3.5 electrons. The AcAcH-CO_2_ catalyst stands out among the different materials, and it shows remarkable performance compared to the other precursors. In this case, the number of electrons transferred at 0.6 V is 3.9, indicating high selectivity, very close to the ideal value of 4, which is the most energetically efficient route for the oxygen reduction reaction. Furthermore, this electrocatalyst reaches a limiting current density of −5.2 mA cm^−2^, a value comparable to that of the commercial material, but using a significantly lower amount of metal, only 10.6 wt% in contrast to 20 wt% of the commercial material.

A clear correlation between the structural features and ORR performance was observed across the different Pd-doped samples. Specifically, samples with thinner carbon fibers and smaller Pd nanoparticles exhibited superior electrocatalytic activity (see Table 1). For instance, the AcAcH sample, which demonstrated one of the best ORR performances, had a fiber diameter of 1.4 ± 0.1 µm and Pd particles averaging 27.2 nm. Similarly, AcAcL, which also showed good activity, exhibited the smallest Pd nanoparticles (24.1 nm) and thin fibers (1.4 ± 0.2 µm). Despite this general trend, the ClH sample achieved the highest ORR activity, as evidenced by its limiting current density and half-wave potential. This exceptional performance can be attributed to its high metal content, even though it exhibited the largest Pd particles and significantly thicker fibers. However, the brittleness of ClH, combined with its elevated Pd loading, limits its practical application as a self-standing electrode in Zn–air batteries, whereas AcAcH is recovered as a flexible carbon cloth with smooth Pd dispersion on the carbon structure, as depicted in the previous section. For this reason, AcAcH sample was selected for further porosity development. For this purpose, the carbonization atmosphere was switched to CO_2_ (sample AcAcH-CO_2_), showing the final material a notable improvement in the electrochemical response, which is attributed to a greater accessibility of the electrolyte in the fiber, as well as a significantly lower resistive behavior.

CO_2_ activation leads to two notable effects. The first, albeit undesirable, is the increase in the size of the Pd nanoparticles. However, this process also triggers a significant benefit: the increase in porosity. This development in porosity considerably improves the diffusion of the electrolyte through the carbon fiber structure, facilitating interaction with the previously inaccessible Pd nanoparticles. Consequently, a remarkable increase in electrocatalytic activity is observed, due to the contribution of these new active sites, as evidenced by the increase in current in the voltammogram and the increase in the current density reached in the LSV.

It is also relevant to note that the polarization curve of this sample shows a steeper slope at the beginning of the reaction, indicating superior reaction kinetics in the oxygen reduction reaction with a half-wave potential of 0.78 V in comparison with the obtained for the AcAcH electrocatalyst of 0.73 V.

To evaluate the enhancement and stability after CO_2_ activation, additional experiments were performed. Initially, the value of the double layer capacitance (*Cdl*) was determined through the analysis of the intermediate slope in the graph of the difference between the anodic (Ja) and cathodic (Jc) currents versus the scan rate (Figures S3 and S4). This calculation was performed at a potential of 0.85 V, specifically selected to avoid the faradic process associated with the reduction in palladium oxide visible in the voltammograms. These carbon fibers have a double layer capacitance of 22.4 mF cm^−2^, significantly higher than the values reported in previous studies [27]. With the current measured and the amount of Pd, we proceeded to calculate the turnover frequency (TOF) of the reaction, resulting in 0.084 s^−1^. This value, derived from the Pd concentrations determined by XPS, exceeds those recorded for all the carbon fibers analyzed in previous studies [27], which underlines the advantages in terms of accessibility of the additional porosity generated in this material by the CO_2_ activation.

The durability of these materials to the ORR was evaluated by LSV test after 500 cycles (see Figure S5). A slight decrease of 5.8% in the limiting current was detected when comparing the curves at 0.4 V; however, the onset potential was unchanged, evidencing the outstanding stability of these fibers for ORR.

In addition, the electrochemical impedance spectroscopy (EIS) results for the AcAcH-CO_2_ electrocatalyst are presented in Figure S6. Here, a noticeable reduction in the resistance of the AcAcH-CO_2_ sample compared to the carbon fiber without metal is observed. After the 500 cycles, a second EIS evaluation was performed, without observing significant changes in the spectrum, which confirms the exceptional electrochemical stability of the materials studied (Figure S7). It must be noted that CO_2_ activation generates mesopores (0.12 vs. 0.03 cm^3^/g in AcAcH, Table 2), which facilitates mass transport and electrolyte diffusion to Pd active sites. This, despite some nanoparticle growth, indicates improved accessibility. Additionally, CO_2_ etching introduces carbon defects that lower charge-transfer resistance and promote electron transfer to Pd, as supported by EIS data and increased TOF values. These defects also help stabilize Pd in its metallic state (Pd⁰), favoring the 4-electron ORR pathway and enhancing kinetics, as reflected in the ORR slope. Finally, the mesoporous structure physically confines Pd nanoparticles, mitigating agglomeration during extended cycling and contributing to long-term durability.

### 3.3. Zinc–Air Battery Performance

**Figure 8:**
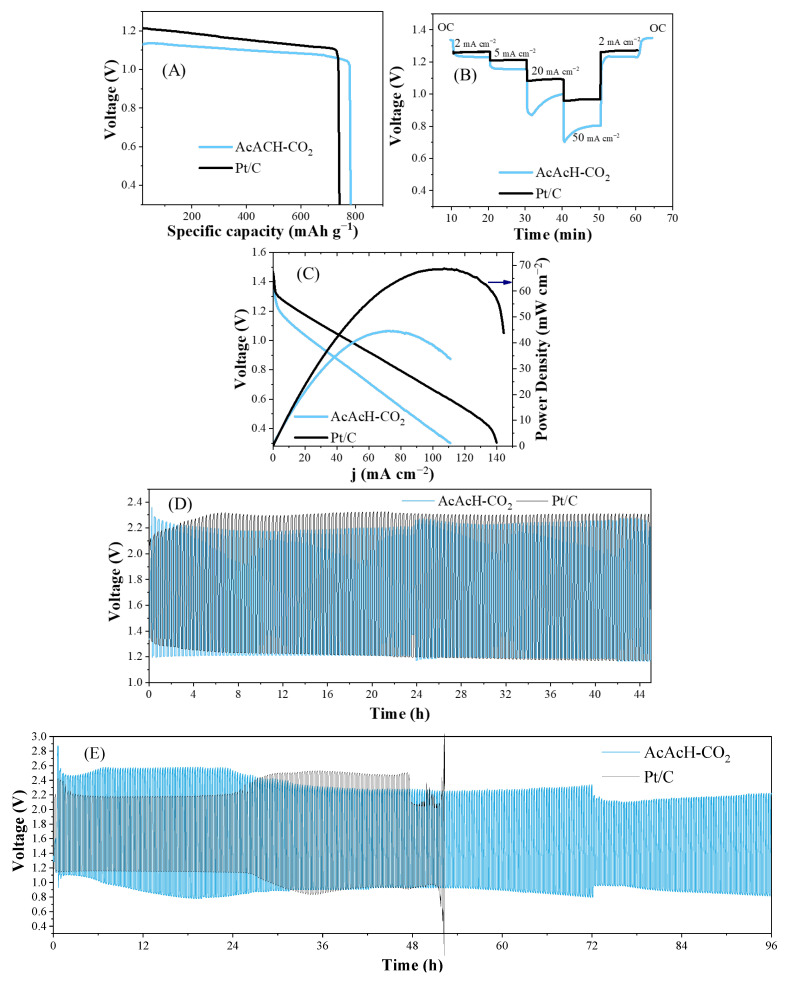
Zinc–air battery performance. (**A**) Galvanostatic discharge curve at 5 mA cm^−2^. (**B**) Discharge curves at current densities from 2 to 50 mA cm^−2^. (**C**) Discharge polarization and corresponding power density curves. (**D**) Charge–discharge cycling performance at 1 mA cm^−2^. (**E**) Charge–discharge cycling performance at 5 mA cm^−2^.

In Figure 8, we evaluate the performance of the AcAcH-CO_2_ electrocatalyst in a zinc–air battery, which was previously shown to be the best electrocatalyst. Figure 8A compares the galvanostatic discharge curve to the commercial material. The results show that this electrocatalyst reaches a capacity of 782 mAh g^−1^, exceeding the 741 mAh g^−1^ of the commercial material. In Figure 8B, we examine the response of AcAcH-CO_2_ electrocatalyst at different current densities during discharge and the Pt/C electrocatalyst is also added for comparison purposes. Its remarkable resistance stands out when supporting high current densities, from 2 mA cm^−2^ to 50 mA cm^−2^, and its ability to maintain constant performance when returning to lower densities of 2 mA cm^2^. At high current densities of 20 and 50 mA cm^−2^, a continuous increase in discharge voltage was observed during the initial stages of operation. This behavior is primarily due to the gradual formation of more efficient oxygen diffusion channels within the porous carbon fiber structure. Additionally, the progressive activation of Pd catalytic sites due to surface restructuring under high current densities, enhances ORR kinetics and reduces overpotential. Localized heating effects may also occur, improving ionic conductivity and accelerate electrochemical reactions. This type of behavior is commonly observed in Zn–air batteries employing porous, binder-free electrodes under demanding current conditions [52,53]. Figure 8C presents the polarization curve obtained with AcAcH-CO_2_ electrocatalyst in which the voltage and the power density are compared with a commercial Pt/C electrocatalyst with 20 wt% of Pt. It reaches a current density of 110 mA cm^−2^ and a maximum power density of 45 mW cm^−2^, values slightly lower than those of the commercial material.

Finally, Figure 8D shows the performance of the AcAcH-CO_2_ electrocatalyst against the charge–discharge test at 1 mA cm^−2^, compared to the commercial material over 45 h. It highlights the need for a lower voltage range between charge and discharge, indicating excellent stability and cyclability of the material. Given this promising initial performance, the material was subjected to more demanding charge–discharge cycles, as shown in Figure 8E, where it was tested at 5 mA cm^−2^. Although the material exhibits a higher overpotential than the commercial catalyst during the first 30 h, a notable improvement in both charge and discharge voltages is observed after this period. Eventually, the material surpasses the performance of the commercial catalyst based on platinum (20 wt.%) and even outperforms a bifunctional commercial catalyst containing 20 wt.% Pt and 20 wt.% RuO_2_ supported on carbon black (Figure S8). Although the bifunctional catalyst initially exhibits lower overpotentials for both charge and discharge, it rapidly degrades after 40 h and fails shortly after 50 h, whereas AcAcH-CO_2_ maintains a lower and more stable overpotential. This enhanced performance is attributed to a better electrolyte penetration into the porous fiber structure, enabling a more efficient contribution of the internal active sites to the battery operation. A similar phenomenon was observed in the cyclic voltammetry measurements, where several cycles were required to achieve a stable voltammogram. Furthermore, after 50 h (when the Pt-based battery breaks down), our material continues to operate. The experiment was stopped after 168 h due to a detected leak in the gas diffusion layer, but as shown in Figure S9, the material maintained its performance throughout this extended testing period. It is also important to note that some abrupt changes in performance were observed during the experiment, coinciding with electrolyte replacement. This suggests that the performance decrease is related to electrolyte degradation and, as observed later, degradation of the zinc foil. This exceptional performance is attributed to the excellent electrical conductivity of the carbon fibers, as illustrated in Figure 6. Additionally, the material can be integrated directly into the system as a self-standing electrode, maintaining its continuous structure. This approach not only simplifies assembly but also opens up new possibilities for research on flexible and self-standing electrodes.

Although zinc–air batteries have been extensively studied, no carbon fiber electrodes derived from lignin, prepared via electrospinning, without the use of any binder, doped with palladium nanoparticles, and directly tested in a battery system have been reported to date. However, studies with similarities in methodology and materials provide useful benchmarks for comparison.

For example, Chen et al. presented wood-based flexible electrocatalysts. The developed material demonstrated excellent performance, sustaining up to 115 h at a current density of 5 mA cm^−2^. Additionally, it achieved a specific capacity of 767 mAh g^−1^ at 5 mA cm^−2^, surpassing the commercial Pt/C+RuO_2_ catalyst, which reached 756 mAh g^−1^ under the same conditions [54].

Other studies have focused on carbon fiber-based electrocatalysts incorporating graphene, tested in zinc–air batteries. These materials achieved a specific capacity of 707 mAh g^−1^, with a charge–discharge cycling stability of over 26 h at a lower current density of 2 mA cm^−2^ [55]. Similarly, carbon fiber paper-based electrodes using cobalt oxides (Co_3_O_4_/CP) have been explored as flexible electrocatalysts for zinc–air batteries. While they demonstrated promising performance, they were only tested at 2 mA cm^−2^ for 60 h, limiting the comparison at higher current densities [56]. Other flexible cobalt-based electrodes have also been studied, where the performance was compared to commercial Pt/C + IrO_2_ materials. Despite being tested at a low current density of 1 mA cm^−2^, the commercial material showed a lifespan of less than 100 h under these conditions. In contrast, the developed cobalt-based electrodes achieved a specific capacity of 770 mAh g^−1^ [57].

Comparing these results with those obtained in our study, our materials present a significant advancement in this field. Operating at a demanding current density of 5 mA cm^−2^, our palladium-doped, self-standing carbon fiber electrodes, not only outperform commercial materials in terms of durability and stability but also exhibit excellent specific capacity. These properties highlight the potential of our material to enrich the research landscape for zinc–air battery applications, combining simplicity of fabrication, a binder-free structure, and outstanding electrochemical performance.

Beyond electrochemical performance, the fabrication strategy exhibits key advantages for scalability. Lignin is a widely available and low-cost biomass byproduct, and the electrospinning method is already used at industrial scale for advanced carbon materials. The CO_2_ activation step employs a safe, inexpensive gas and can be directly integrated into the carbonization step without additional chemical treatments. Remaining challenges include the cost of Pd and fiber handling at scale; however, these can be mitigated through Pd-efficient deposition strategies and integration with flexible current collectors. The binder- and additive-free design simplifies electrode assembly, supporting the potential transition toward industrial application.

## 4. Conclusions

In summary, through the electrospinning technique, carbon fibers were obtained from various palladium precursors under different preparation conditions, using lignin as a carbon feedstock, without using any structure stabilizing agent. This study confirmed that highly active ORR electrodes consisting of Pd-containing carbon microfibers shaped into carbon cloths can be easily obtained by mixing the salt precursors into the lignin-ethanol solution. The preparation conditions for optimum performance in the ORR test under alkaline electrode were attained using (i) platinum acetylacetonate as palladium precursor, (ii) a Pd salt/lignin weight ratio of 0.015 in the starting solution, (iii) air thermostabilization step at 200 °C with a holding time of 48 h and (iv) carbonization at 900 °C. Consequently, homogeneous distribution of Pd^0^ nanoparticles within the carbon fiber structure along with a well-developed micropore structure, Pd loading of ca. 5% and appropriate structural order, are obtained. Furthermore, it was shown that when the thermal treatment is carried out in CO_2_ atmosphere, the surface area increases and additional porosity development is obtained, improving the accessibility of the electrolyte to the palladium active sites. As a result, a significant improvement in the reaction kinetics was achieved, reaching limiting current densities comparable to those obtained with the commercial material, with the advantage of requiring a lower metal load (10.6% vs. 20%) and showing great selectivity towards the 4-electron path.

Furthermore, the continuous structure of the fibers allowed them to be quickly and easily assembled as a self-standing electrode in the zinc–air battery, where they demonstrated exceptional performance compared to the commercial Pt/C material. They reached a higher capacity of up to 782 mAh g^−1^. The results from cycling tests at a higher current density of 5 mA cm^−2^ further validated the outstanding durability, stability, and long-term performance of these electrodes, surpassing those of commercial materials tested under the same conditions. This demonstrates the robustness of the developed materials and their suitability for demanding practical applications. This type of high-performance flexible electrode opens the door to a wide range of applications in flexible electronics and represents a significant advance in the research and development of advanced materials for energy technologies of the future.

## Figures and Tables

**Table 1:** Stabilization, carbonization and total preparation yields of carbon fibers production. Average carbonized fibers size and Pd particle size (calculated from SEM and TEM images, respectively).

Sample	Yield (wt%)			Diameter Carbon Fiber	Pd Particle Size
	Stabilization	Carbonization	Total	(µm)	(nm)
No Pd	71	37	27	1.6 ± 0.2	-
ClL	65	36	23	1.4 ± 0.3	34.5
ClH	55	30	16	18.0 ± 0.8	85.4
AcL	50	48	24	1.6 ± 0.4	37.8
AcH	52	45	24	1.6 ± 0.2	59.7
AcAcL	43	42	18	1.4 ± 0.2	24.1
AcAcH	37	53	20	1.4 ± 0.1	27.2
AcAcH-12 h	61	48	29	2.1 ± 0.3	75.4
AcAcH-CO_2_	37	36	13	1.3 ± 0.1	53.3

**Table 2:** Summary of the textural parameters obtained from N_2_ adsorption–desorption isotherm and mass surface concentration derived from XPS analyses and Pd content, calculated by TG analysis.

Sample	Ads-Des N_2_			XPS (wt%)			Pd Content
	A_BET_ (m^2^/g)	V_meso_ (cm^3^/g)	V_micro_ (cm^3^/g)	C	O	Pd	(wt%)
No Pd	865	<0.01	0.34	95.0	4.5	-	-
ClL	795	0.06	0.31	88.3	7.2	4.5	1.6
ClH	750	0.38	0.22	74.1	12.9	13.0	6.2
AcL	640	0.02	0.24	82.0	9.8	8.2	2.9
AcH	615	0.01	0.23	78.2	10.6	11.2	5.9
AcAcL	640	0.01	0.24	92.0	6.5	1.5	2.5
AcAcH	655	0.03	0.25	87.4	8.4	4.2	5.5
AcAcH-12 h	595	0.02	0.23	91.7	7.4	0.9	3.6
AcAcH-CO_2_	1460	0.12	0.67	88.7	8.2	3.1	10.6

**Table 3:** ORR electrochemical parameters in alkaline electrolyte.

Electrocatalyst	E_onset_ (V) (at j = −0.1 mA cm^−2^)	n (at 0.6 V)	j (mA cm^−2^) (at 0.4 V)	Half-Wave Potential (V)
ClL	0.84	3.8	−2.3	0.70
ClH	0.91	4	−3.7	0.78
AcL	0.87	3.8	−3.2	0.73
AcH	0.87	3.8	−2.3	0.73
AcAcL	0.79	4	−1	0.63
AcAcH	0.89	3.9	−3.5	0.74
AcAcH-CO_2_	0.86	3.9	−5.2	0.78
Pt/C	0.98	3.9	−5.7	0.86

## Data Availability

Data are contained within the article and Supplementary Materials.

## Supplementary Materials

The following supporting information can be downloaded at: https://www.mdpi.com/article/10.3390/molecules30122487/s1, Figure S1: HRTEM images of: (A) ClH, (B) AcH, (C) AcAcH, and (D) TEM mapping image for the carbon fiber electrocatalyst AcAcH-CO_2_. Figure S2: Pd3d XPS spectra for the Pd fibers. Table S1: Selected first-order Raman spectra deconvolution parameters. Figure S3: Cyclic voltammograms at different scan rates, for AcAcH-CO_2_. Figure S4: Ja and Jc vs. scan rate plot for the AcAcH-CO_2_ electrocatalyst. Figure S5: Linear sweep voltammograms for the AcAc-H-CO_2_ electrocatalyts before and after the stability test of 500 cycles. Figure S6: Impedance spectra for the AcAcH-CO_2_ carbon fiber inks using a N_2_-saturated 0.1 M KOH, at 0.2 V vs RHE, frequencies from 6000 to 0.1 Hz. Figure S7: Impedance spectra for AcAcH-CO_2_ carbon fiber ink before and after the stability test with 500 cycles. Figure S8. Charge discharge cycling performance comparison at 5 mA cm^−2^ for Pt/C-RuO_2_ and AcAcH-CO_2_. Figure S9: Charge discharge cycling performance at 5 mA cm^−2^ for 170 hours.
