# Supplementary material for: Self-Standing Carbon Fiber Electrodes Doped with Pd Nanoparticles as Electrocatalysts in Zinc–Air Batteries
Source: Molecules. 2025 Jun 6;30(12):2487. doi: 10.3390/molecules30122487 (PMC12196446; doi:10.3390/molecules30122487)
Supplement: Supplementary file 1 [file molecules-30-02487-s001.zip › molecules-3632686-supplementary.pdf]

Self-standing carbon fiber electrodes doped with Pd nanoparticles as electrocatalysts in Zinc-air battery

Cristian Daniel Jaimes-Paez[1], Miguel García-Rollán[2], Francisco José García-Mateos[2], Ramiro Ruiz-Rosas[2]*, Juana M. Rosas[2], José Rodríguez-Mirasol[2], Tomás Cordero[2], Emilia Morallón[1]* and Diego Cazorla-Amorós[3]

Electrocatalyst preparation details

The electrocatalyst solutions were prepared using lignin (Alcell®, obtained through the organosolv process), ethanol, and three types of palladium precursor salts: palladium acetate (Ac), palladium acetylacetonate (AcAc), and palladium chloride (Cl). Lignin was mixed with ethanol in a 1:1 mass ratio. Different Pd/lignin weight ratios (0.0075 and 0.015) were used, corresponding to Pd nominal weight concentrations of 2.5 wt% (L) and 5 wt% (H) in the resulting carbon fibers.

The lignin/ethanol/Pd salt solutions were stirred at 250 rpm and maintained at 60 °C overnight before electrospinning. The electrospinning process was performed using a coaxial system with an applied voltage range of 15 to 18 kV and a 25 cm injector-collector distance. The lignin solution flow rate was adjusted to 1–1.2 mL h^{-1} through the inner needle, while ethanol flowed at 0.10–0.15 mL h^{-1} through the outer needle. When using palladium chloride at the highest Pd/lignin ratio, the flow rates were increased to 6 mL h^{-1} and 0.6 mL h^{-1} for the inner and outer needles, respectively, with a voltage increase to 28 kV for stable electrospinning.

The thermostabilization process in air involved a gradual temperature increase from 60 °C to 200 °C at 5 °C h^{-1}, with holding times of 12 or 48 hours. Carbonization was conducted under nitrogen at a heating rate of 10 °C min^{-1} up to 900 °C, forming the final carbon fibers. The impact of a CO_2 atmosphere during thermal treatment at 900 °C for 2 hours was also evaluated.

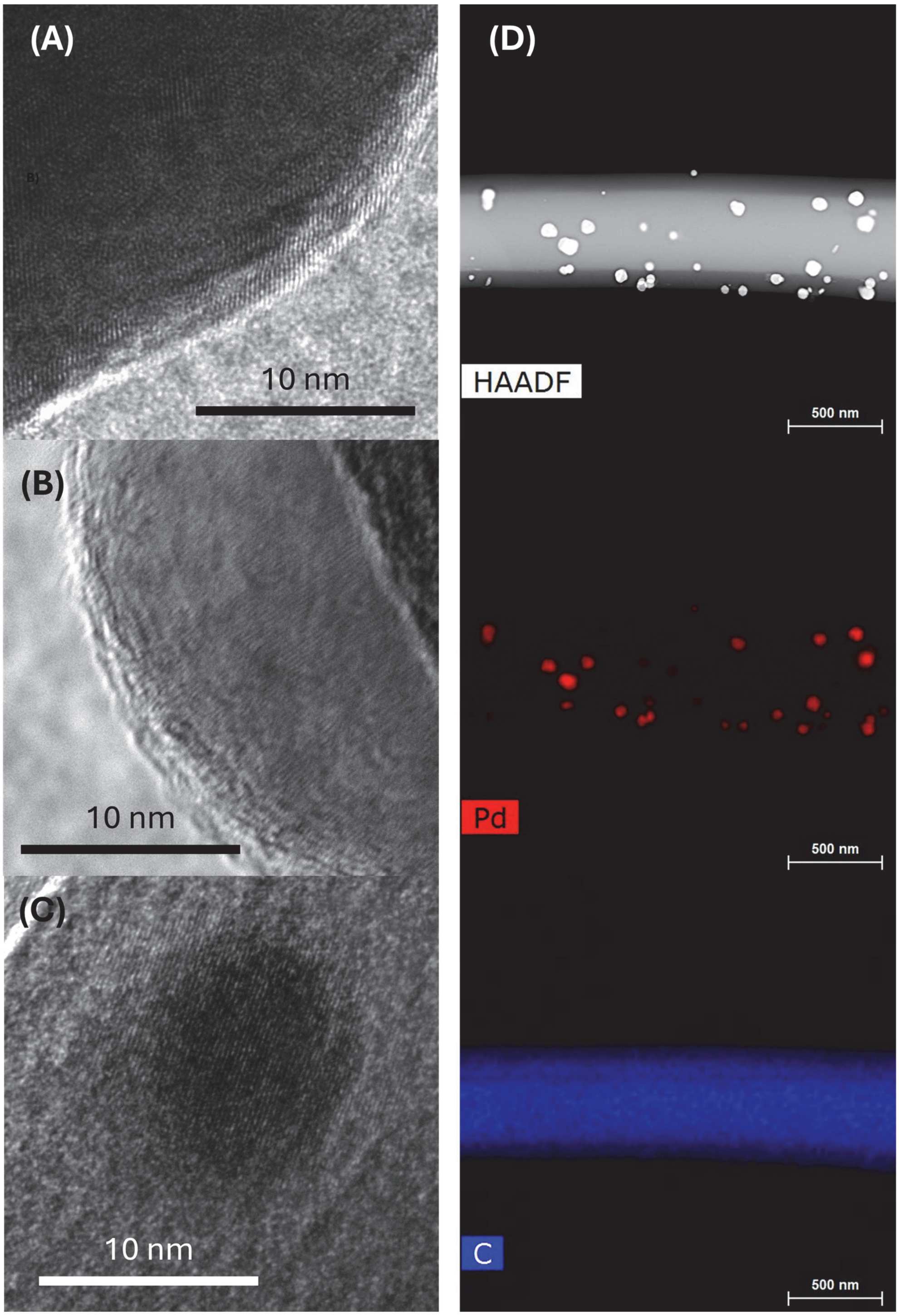

Figure S1. HRTEM images of: (A) ClH,(B) AcH, (C) AcAcH, and (D) TEM mapping image for the carbon fiber electrocatalyst AcAcH-CO_2.

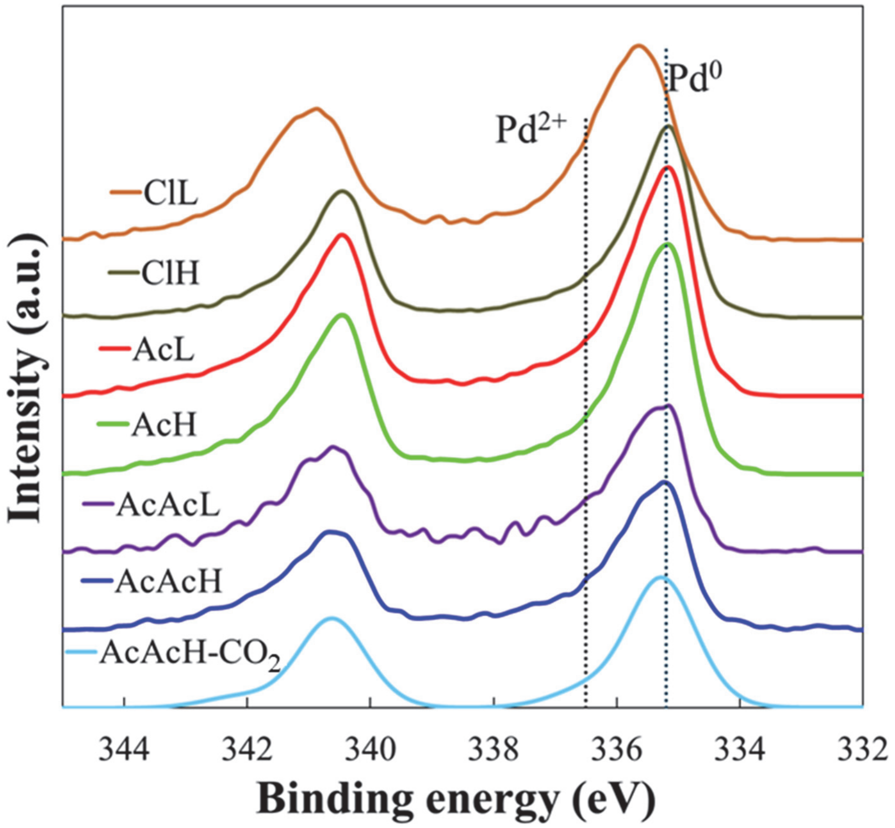

Figure S2. Pd3d XPS spectra for the Pd fibers.

Table S1. Selected first-order Raman spectra deconvolution parameters.

Sample	I_G/I_T (%)	$Position_D$ (cm^{-1})	$FWMH_D$ (cm^{-1})	I_{1170}/I_T (%)	$Position_G$ (cm^{-1})	$FWMH_G$ (cm^{-1})	I_G/I_D
No Pd	29.5	1350	153.2	14.5	1588.1	86.6	1.02
ClL	27.9	1350	162.9	12.5	1590.0	88.4	1.02
ClH	29.9	1350	158.9	8.1	1590.0	86.6	0.98
AcL	26.3	1350	172.0	9.7	1590.0	91.9	1.02
AcH	28.9	1350	164.3	11.3	1590.0	109.0	1.02
AcAcL	26.5	1340	161.7	9.7	1590.0	89.7	1.03
AcAcH	29.7	1333	154.3	11.1	1590.0	88.4	1.01
AcAcH-CO_2	24.1	1329	153.0	9.7	1590.0	70.2	0.98

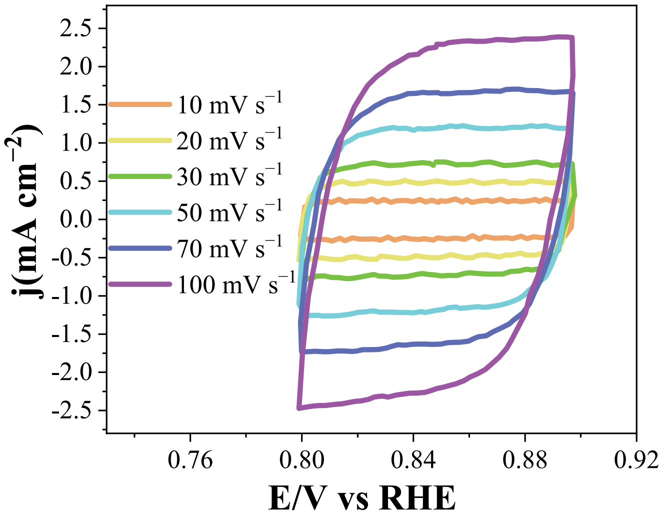

Figure S3. Cyclic voltammograms at different scan rates, for AcAcH-CO_2.

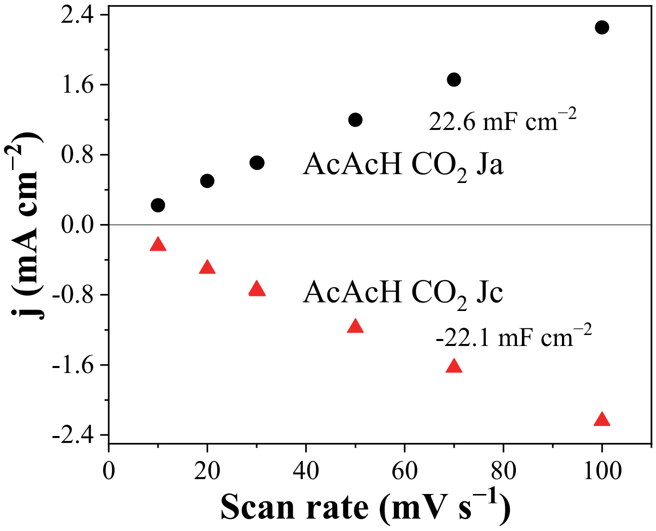

Figure S4. Ja and Jc vs. scan rate plot for the AcAcH-CO_2 electrocatalyst.

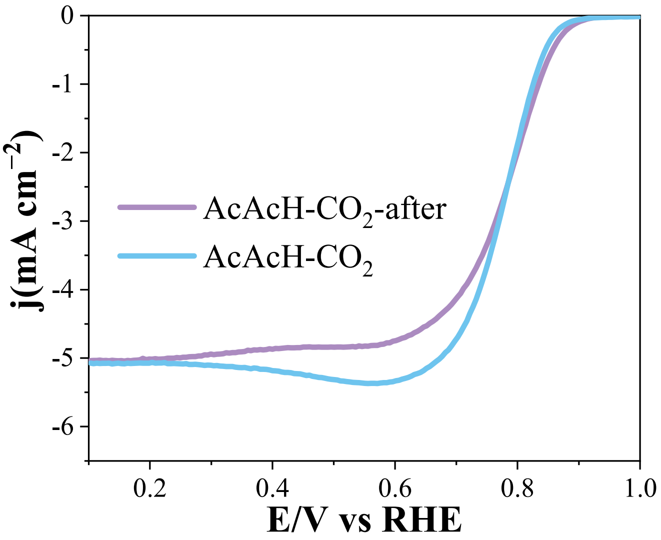

Figure S5. Linear sweep voltammograms for the AcAcH-CO_2 electrocatalysts before and after the stability test of 500 cycles.

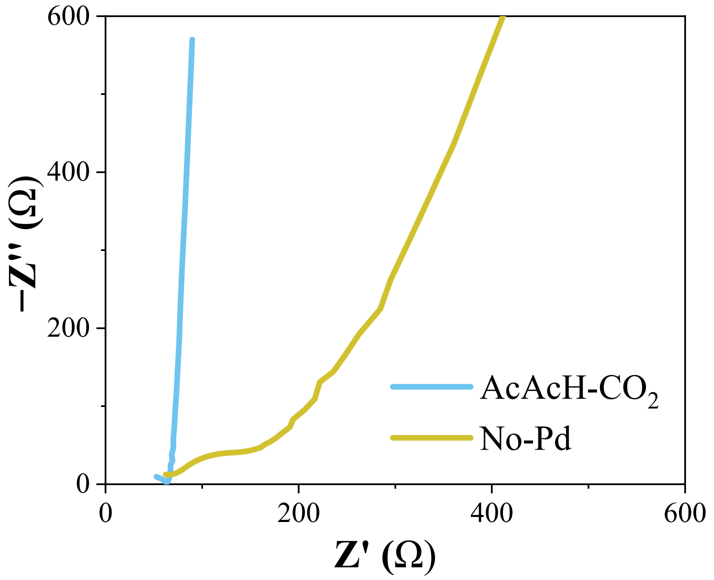

Figure S6. Impedance spectra for the AcAcH-CO_2 carbon fiber inks using a N_2-saturated 0.1 M KOH, at 0.2 V vs RHE, frequencies from 6000 to 0.1 Hz.

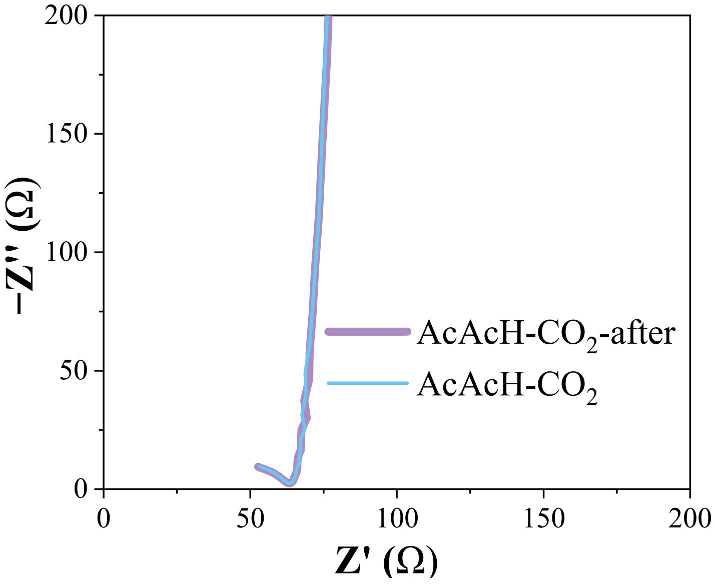

Figure S7. Impedance spectra for AcAcH-CO_2 carbon fiber ink before and after the stability test with 500 cycles.

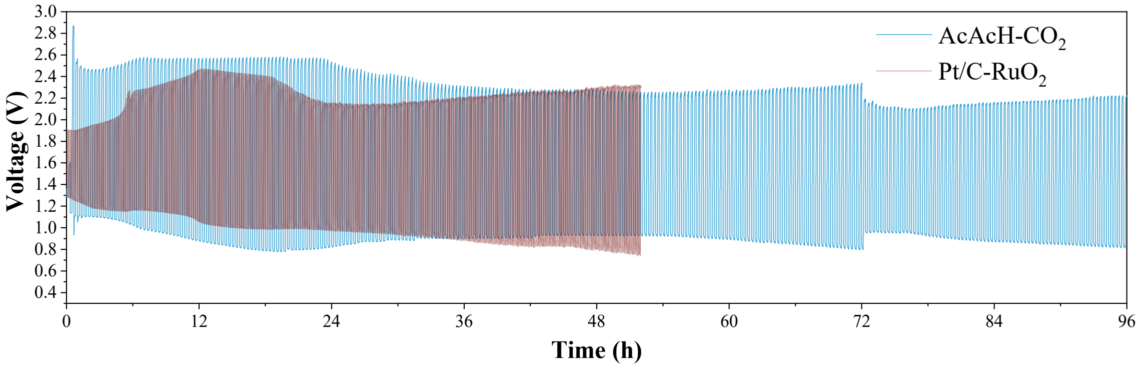

Figure S8. Charge discharge cycling performance comparison at 5 mA cm^{-2} for Pt/C-RuO_2 and AcAcH-CO_2.

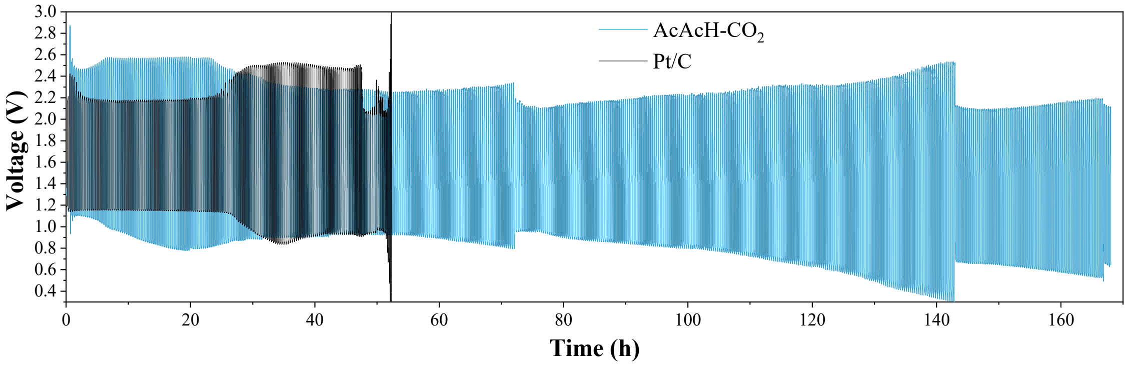

Figure S9. Charge discharge cycling performance at 5 mA cm^{-2} for 170 hours.
